# Improving health worker motivation and performance to deliver adolescent sexual and reproductive health services in the Democratic Republic of Congo: study design of implementation research to assess the feasibility, acceptability, and effectiveness of a package of interventions

**DOI:** 10.1080/16549716.2021.2022280

**Published:** 2022-01-20

**Authors:** Sheri Bastien, Erin Ferenchick, Symplice Mbola Mbassi, Marina Plesons, Venkatraman Chandra-Mouli

**Affiliations:** a Department of Public Health Science, Norwegian University of Life Sciences, Ås, Norway; b Cumming School of Medicine, Department of Community Health Sciences, University of Calgary, Calgary, Canada; c Technical Advice and Partnership Department, Geneva, Switzerland; d Regional Office for Africa, World Health Organization, Geneva, Switzerland; e Department of Sexual and Reproductive Health and Research, World Health Organization, Geneva, Switzerland

**Keywords:** Adolescent sexual and reproductive health, health worker training, collaborative learning, implementation research, heath worker motivation, health worker performance

## Abstract

During its last funding cycle from 2018–2020, the Global Fund in collaboration with the Ministry of Health, World Health Organization, and implementing partners Cordaid and Santé Rural (SANRU), implemented a multi-sectoral, contextualized approach to improve the sexual and reproductive health of adolescent girls and young women in two regions in the Democratic Republic of the Congo, which included community-based, school-based and health facility-based actions. This implementation research focuses on the health-facility component. The objective of this research is to evaluate the feasibility, acceptability, and effectiveness of a package of interventions to improve health workers’ knowledge, skills, and attitudes in providing sexual and reproductive health services to adolescents, whilst concomitantly creating an enabling work environment for building health workers’ motivation. The package includes a combination of job descriptions, training and refresher training, desk reference tools, and collaborative learning. The package did not focus on improving amenities, providing or repairing equipment, or providing medicines and supplies. The underlying theoretical framework informing the project and the implementation research draws from Social Network Theory, Diffusion of Innovations and Normalization Process Theory. Qualitative and quantitative process and outcome data from in-depth interviews and focus group discussions with health workers and health managers, field notes, monitoring reports, costing sheets, and health worker surveys, adolescent mystery client assessments, and exit interviews with adolescents will be collected as part of a time-series study. The findings from this implementation research will be utilized to inform future adaptations and/or scale-up of the package of interventions to improve health worker motivation and performance in the Democratic Republic of the Congo and elsewhere. The findings will also contribute to advancing the use of theoretical approaches within the field of implementation research.

## Background

Promotive, preventive, and curative health services are an important component of a package of interventions to improve the sexual and reproductive health (SRH) of adolescents. A crucial ingredient of a high quality, friendly, and responsive health service is a capable and empathic health worker. Yet inadequate competency (i.e. knowledge and skills to carry out a task), judgmental attitudes, and low motivation leading to poor clinical and interpersonal performance are widespread. This has negative implications for the provision and utilization of health services, especially SRH services, for adolescents [1]. Delivering accessible, high quality adolescent sexual and reproductive health (ASRH) services that meet the needs and preferences of adolescents could lead to increased uptake of services and improved sexual and reproductive health outcomes.

Evidence suggests that factors operating at the individual, institutional and social levels affect health worker performance, and that these factors need to be addressed to improve health worker competencies, attitudes (including biases) and motivation [2]. Likewise, it suggests that attention to the context is crucial, and that local conditions must be addressed by interventions. In terms of improving competencies, evidence suggests that training alone is insufficient to lead to sustained improvements in health worker performance, and that comprehensive approaches that combine interactive and participatory training, job aids, supportive supervision and collaborative learning are more effective than piecemeal approaches in building competencies, positive attitudes and motivation and thereby improving performance [3,4]. However, in many instances training, often one-off training that generally uses didactic methods, is the only performance-improvement approach that is used in large-scale programmes [5].

Similarly, much attention is given to the importance of financial incentives in sustaining health worker motivation and performance. Whilst financial incentives play a role in sustaining health worker motivation and performance, a systematic review that assessed the effectiveness of strategies to improve health-care provider practices in low- and middle-income countries found that interventions using group problem solving plus training had a larger effect size than those addressing financial incentives [2]. Likewise, a study in India found that health worker motivation and performance can be improved through non-financial incentives, such as teamwork and recognition [6]. Finally, a study in Benin and Kenya found that non-financial incentives and human resource management tools to provide an enabling environment play an important role in increasing health worker motivation [7].

While our knowledge of efficacious and effective interventions to improve ASRH has improved, gaps remain in our knowledge of how to deliver these interventions effectively at scale in resource-constrained settings, while ensuring quality and equity. It is clear that interventions that have shown to be effective in research or small-scale project contexts have, in many cases, not been effective when implemented in large-scale programmes. However, it is not always clear why this is so and what can be done to remedy this. Implementation research can help us to understand how and why implementation is going well or not well, and to test approaches to improve the situation in specific contexts [8].

In this manuscript, we describe the broader context in which the project was implemented in, with a focus on the implementation research on the health-facility component which aims to assess the feasibility, acceptability, and effectiveness of a package of interventions to improve health worker motivation and performance using a time-series design. The first and second rounds of data collection are planned to be completed by December 2021, with additional rounds planned in 2022. Here we describe the protocol for the implementation research. Forthcoming publications will describe the implementation process and findings from the study.

### Context

In the Democratic Republic of the Congo (DRC), adolescents in general and adolescent girls in particular are at risk of poor sexual and reproductive health outcomes for a host of reasons at the individual, community, and structural levels. Early sexual debut, unprotected sexual activity, transactional sex, and violence, including sexual violence coerced sex are widespread [9,10]. The HIV epidemic in DRC is generalized with a prevalence of 0.7% in the general population (15 to 49 years) and notably higher among women (1.1%) than men (0.4%) [11]. Among young women, the prevalence is 0.4% and among young men it is 0.2%. The low HIV prevalence at the national level in the DRC in comparison to neighboring countries masks geographic disparities within the country with prevalence higher in urban areas. Further, the proportion of ever-partnered women aged 15–49 years who have reportedly experienced intimate partner physical and/or sexual violence at least once in their lifetime is 51% [10], although even this figure is likely an underestimate given that a substantial proportion of survivors do not report or seek care and support.

### Objectives of the overall project

To address these issues and challenges, the Global Fund to Fight AIDS, Tuberculosis and Malaria (Global Fund) in collaboration with the Ministry of Health, World Health Organization, and implementing partners Cordaid and Santé Rural (SANRU), implemented a multi-sectoral, contextualized approach to improving the SRH of adolescent girls and young women (AGYW) in two regions in DRC (Kasai Oriental and Kinshasa), which included community-based, school-based and health facility-based actions. The objectives of the project were: i) to increase the knowledge and understanding of SRH, HIV, human rights and GBV among AGYW; (ii) to reduce GBV cases in the school and the community; and iii) to improve the provision of adolescent-responsive health services in terms of both quality and access. The selection criteria for the geographical distribution of the study sites participating in the project included HIV prevalence rates among AGYW aged 15–24 years, as well as the presence of health services already being supported by the GFATM through existing grants. Intervention activities within health facilities focused on 3 health zones in each of the two regions were intended to include coordination strengthening, supportive supervision, the provision of in-service training, job aids, and collaborative learning sessions. They aimed to build the capacity of health workers to respond to adolescent clients more effectively and with sensitivity and empathy, while ensuring that health workers have access to necessary supports (i.e. health products financed out of the grants) to enhance health worker performance through an enabling work environment.

### Formative phase (2017-2019)

In the first phase of the project, formative research was undertaken to assess the quality of health service provision to adolescents and to identify actions to build on strengths and address areas of weakness. A baseline assessment was carried out in 30 health facilities (5 facilities in each of the 3 health zones in Kasai Oriental and Kinshasa, respectively) in November 2017, consisting of semi-structured interviews with health workers and adolescent clients, as well as systematic observation of health facilities [12,13]. The reports from these assessments note that facilities had basic equipment and essential medicines, and that they were staffed and functioning staff in place. While the findings in both Kasai Oriental and Kinshasa indicate that the majority of health workers had not been trained on the provision of SRH services to adolescents, health workers reported that they respect adolescents and young people, that they feel confident in providing services to them, and that they do not judge them. However, the reports highlight that none of the facilities offered a complete health service package or educational materials tailored to the needs of adolescents; or specific activities related to this group. In addition, almost all facilities lacked preferential pricing for adolescents and young people. Finally, adolescent clients themselves reported dissatisfaction with for example the length of waiting time to receive services [12,13].

In terms of actions, an initial two-day health worker training in Kinshasa was conducted in 2017 by the World Health Organization Regional Office for Africa (WHO AFRO) and subsequently in Mbuji-Mayi, Kasai Oriental in early 2018, with a focus on developing a shared understanding of the period of adolescence, adolescents’ specific health needs, and how to deliver SRH services to adolescents with dignity and respect. Following this, desk reference tools were distributed to them.

In March 2018, WHO-AFRO facilitated a four-day meeting to sensitize the Ministry of Health, specifically the National HIV/AIDS Control Programme (Programme National de Lutte contra le VIH/Sida, PNLS) and the National Adolescent Health Programme (Programme National de Santé de l’Adolescent, PNSA), implementing partners, and key stakeholders to the collaborative learning approach. This meeting was also used as the forum for validating a draft operational manual prepared by WHO AFRO for the collaborative learning model to be piloted in the project. Collectively the participants reviewed and adapted this draft, ultimately developing a national ‘Collaborative Learning Guide for Improving the Performance of Health Service Providers’ specific for the context of DRC.

Subsequently, in April 2018, a five-day workshop to train facilitators from Kinshasa and Kasai Oriental on the collaborative learning approach was organized by the Ministry of Health under the leadership of PNLS and PNSA, with the financial support of CORDAID, the primary recipient of the GFATM grant. WHO AFRO provided the technical support and facilitated the workshop and the GFATM provided additional support. The sessions were attended by 30 participants across all levels of the health system from six target health zones in this project with the intention to form a team of facilitators comprised of experts at the national and provincial levels, as well as members of the health zone management teams [14]. This pool of facilitators with expertise in collaborative learning strategies and ASRH has been responsible for supporting the collaborative learning sessions with health workers and managers from the identified health facilities. In addition, this pool of facilitators includes national experts to generate ownership and support systems to contribute to sustainability. Between June 2018 and May 2019, 32 collaborative learning sessions were arranged by Cordaid and PNLS, with logistical support from Réseau National des ONG pour le Développement de la Femme (RENADEF), a local civil society organization, and technical support from the WHO and the Global Fund. During 2019 and 2020 there were 56 collaborative learning sessions held in Kinshasa and Mbuji-Mayi. There are plans for additional sessions to take place during 2021–2022 with funding from the WHO.

Alongside efforts to put in place evidence-based and scalable approaches with proven health and social benefits, this implementation research is being undertaken in order to understand how to tailor and deliver interventions that have shown to be effective in research studies and pilot projects. It thus presents a unique opportunity to address the pressing need for evidence in the area of improving health worker knowledge, skills, and attitudes and how this influences their motivation and performance in relation to the provision of SRH services to adolescents in this context.

### The package of interventions

A package of interventions to enhance health workers’ performance, tailored to the local context, was targeted primarily to health workers, but also to some extent involving health managers at participating health facilities and the health zone management team. This package draws on the available evidence of effective interventions, including a combination of job descriptions, training related to adolescence and adolescent health issues, quality adolescent and youth friendly health services, including refresher training, monitoring and desk reference tools and collaborative learning [3]. In addition, a platform was created for health workers to contribute to ongoing discussions on how to improve working conditions, which aimed to contribute to increased motivation and sustainability of efforts to strengthen the quality of services. Protocols for training, refresher training, and collaborative learning, which are aligned with existing PNSA/DRC standards as well as WHO quality standards and job descriptions, were developed. The package of interventions did not focus on improving amenities, providing or repairing equipment, or providing medicines and supplies.

An overview of the components of the package of interventions to improve health worker competencies, attitudes, motivation, and performance at the health facilities is provided below. In addition to these components, routine supervision was regularly provided by the Ministry of Health and on occasion with WHO AFRO, CORDAID and RENADEF. Despite early intentions of the project to strengthen routine supervision, this aspect was not fully realized.

An in-depth overview of the collaborative learning approach is provided in Box 1 below.

**Box 1.** Overview of the collaborative learning approach

Collaborative learning is an evidence-based approach which brings together groups of health workers and health managers to participate in facilitated group discussions to share and to learn from and with each other, and to jointly identify, define and solve problems, establish norms of good practice, and foster a commitment to continuous learning. The overall objective of collaborative learning in the context of adolescent health is to better equip health workers with the skills to provide quality services tailored to the needs of adolescents and youth and in an enabling environment that ensures respect, protection of their rights to information, privacy, confidentiality, non-discrimination, without judgment and with accountability. The specific objectives are to: •Improve the quality of delivery of adolescent and youth-friendly health services across the health system; •Support health training for the provision of health services suitable for adolescents and young people (acceptable, accessible, equitable, appropriate and effective and affordable); •Strengthen the community’s referral and counter-referral systems to care structures; •Improve the motivation, accountability, and performance of health service providers for the sustainability of achievements; and •Increase the use of health services by adolescents and youth in the community, in schools and care settings. Additional evidence is needed to determine the impact of collaborative learning within the field of adolescent health. However, promising findings from a study conducted in Moldova indicate that the approach was both feasible and acceptable in that context, and that it may contribute to efforts to improve quality of care and youth-friendliness and, increased utilization of health services [28].

### The implementation research at the health-facility level

This implementation research is nested within the Global Fund supported project and focuses on the health facility-based actions. The objectives of the present study are to evaluate the feasibility, acceptability, and effectiveness of a package of interventions to:

(a) improve health workers’ knowledge and skills in providing SRH services to adolescents and to build positive attitudes regarding the provision of SRH services to adolescents; and

(b) create an enabling work environment for building health workers’ motivation to apply their competencies and positive attitudes towards the provision of SRH services for adolescents.

Together, these actions are intended to improve and sustain improvements in health workers’ performance.

### Theoretical framework

Public health interventions tend to be more effective if they are grounded in social and behavioural science theory, which may help predict or explain the pathway to a desired outcome [15]. Within this study, the specific focus on the collaborative learning approach to promote group problem solving and improve health worker competencies, attitudes, practices, and motivation concerning the provision of SRH services to adolescents draws on a cluster of interrelated theories: Social Network Theory [16], Diffusion of Innovations [17,18], and Normalization Process Theory [19]. Each of these theories contributes to understanding how attitudes, norms and practices become embedded in different social settings, including health facilities.

Social Network Theory (SNT) focuses on the structure of relations among individuals with regard to their network and the impact this has on attitudes and practices from the individual to the collective and systems levels. The density and distribution of ties are considered important to understand the spread of information and influence according to the theory, as is social support and the extent to which contacts within the network are homophilous. Within implementation science, SNT can be useful for understanding a programme’s adoption, implementation, and sustainability. From this perspective, it is essential to consider three interrelated aspects of an intervention or programme: partnerships between researchers, practitioners, and the wider community, as well as stakeholders such as policy makers; the characteristics of those who implement interventions as well as the recipients; and the wider context in which the intervention is implemented [20].

Diffusion of Innovations also focuses on social networks and is most often used to investigate the adoption or rejection of new innovations or practices, especially the rate of adoption or rejection and how innovations diffuse through social systems to influence social norms [18]. Diffusion is characterized by five stages: knowledge, persuasion, decision, implementation, and confirmation. This theory suggests that innovations typically spread following an ‘S’ shaped curve, whereby uptake by early adopters is eventually followed by the majority, at which point the innovation becomes commonplace.

Normalization Process Theory (NPT) focuses on mechanisms that influence how a new norm or intervention can become embedded and normalized in everyday practice in institutional and other social contexts and settings, and which factors enable or hinder this process [19]. A recent systematic review of the use of NPT in complex healthcare interventions found that it can be useful in all programming phases, from intervention development and implementation planning, to evaluation of complex implementation processes [21]. According to NPT, practice is routinely integrated into social contexts as a result of the individual and collective work done in connection with the implementation [21]. Within health care settings, implementation of interventions is highly complex due to organizational structures and how they interface with the social contexts in which they are embedded. NPT can be useful for mapping and explaining the relationship between actors, norms, practices, and contexts in relation to the underlying mechanisms of implementation processes.

These three theories are particularly relevant to this study given their emphasis on:

- the importance of communication in social networks (in the context of this study, through group problem solving and collaborative learning among colleagues), to catalyse change,
- the role of modelling, opinion leaders and imitation of others who have successfully adopted newly introduced practices, and,
- the necessity of an enabling environment and organizational culture that is supportive of evidence-based approaches such as collaborative learning, and
- the tailoring of approaches to suit the cultural, economic, political, and social context of the setting.

This study aims to build knowledge and skills and change potentially deep-seated attitudes among health workers through the introduction of the package of interventions, and particularly the collaborative learning approach. Given this, drawing on this cluster of theories both strengthens and guides the implementation research at all phases, from inception through to analysis of the role of the intervention components in shifting practices and norms, as well as the nature and extent of the impact of the intervention components on the provision of SRH services to adolescents.

### Study setting

As described above, the health facility-based component of the project is being implemented in 3 health zones in both Kinshasa and Kasai Oriental. Kinshasa is the country’s capital city and is one of the continents fastest growing cities, with an estimated population of 14,970,000, whereas Mbuji-Mayi, the capital city of Kasai Oriental, has approximately 2,643,000 inhabitants [22]. Kinshasa is linguistically diverse with inhabitants speaking French and other local languages such as Lingala, Kikongo, Tshiluba, and Swahili. Kinshasa’s main economic activities include food processing, manufacturing, construction, and provision of services, and it is an important logistical and transportation hub within Africa. Kasai Oriental is also linguistically and culturally diverse, and the socio-economic setting in the province is influenced by the presence of a diamond industry, which provides employment for a large segment of the population.

## Study design and methods

The study is based on a mixed-methods time-series design. As noted above, a package of interventions is being implemented, and ongoing collaborative learning sessions will take place through 2022. An overview of the package of interventions is provided in Table 1 below. Also mentioned previously, the first two rounds of data collection are planned to take place by the end of December 2021, with 3–4 additional rounds of data collection planned in 2022. The package of interventions will be assessed through process and outcome evaluations. The University of Kinshasa is leading the process and outcome evaluation (2021–2023), with technical support from the WHO. This section describes the implementation research objectives, research questions and data collection methods that will be common across each round of data collection, with an overview provided in Table 2 below.

**Table 1.** The package of interventions

Intervention component	Description and implementation plan	Potential benefits
Jobdescriptions	Job descriptions that set out functions of health managers and workers, in line with existing PNSA documents. The competencies required to carry out the functions are clearly stated and in line with national standards and the WHO ‘Core competencies in adolescent health and development for primary care providers’ (WHO, 2015).	Improved clarity around objectives, responsibilities, authority and lines of accountability in relation to adolescent-responsive SRH/HIV services.
Training	Competency-based in-service training for health managers and workers with a focus on the provision of SRH, including GBV, services to adolescents. Training is followed up with periodic retraining.	Improved knowledge and skills in providing adolescent-responsive SRH/HIV services.
Job aids	Desk reference tools to assist health workers in providing high quality SRH/HIV services to adolescents.	Assurance that everyday work is in line with Standard Operating Procedures.
Collaborative learning	Collaborative learning, including sharing of information with peers and learning from and with them. Support for group problem identification and solving.	Learning from others’ experiences and motivation. A cordial and supportive social environment.

**Table 2.** Overview of objectives, research questions, and data collection methods

Objectives	Evaluation/research questions	Data collection methods and participants
To evaluate the feasibility, acceptability, and effectiveness of a package of interventions to: a) improve health workers’ knowledge and skills in providing SRH services to adolescents and judgment on where/when/how to apply their them (together contributing to their competence), and to build positive attitudes regarding the provision of SRH services to adolescents; and b) create an enabling work environment for building health workers’ motivation to apply their competencies and attitudes for SRH services for adolescents.	How did the health managers perceive the package of interventions? How did they perceive each component of the package, and especially the collaborative learning sessions? (in terms of meeting the needs of health workers with respect to feasibility, and in terms of acceptability)	In-depth interviews with health managers. Reports of collaborative learning sessions, supervision, and monitoring reports. Field notes.
	How much did the delivery of the intervention package cost?	Costing sheets.
	How did the health workers perceive the package of interventions? How did the health workers perceive each component of the package of interventions, and especially the collaborative learning sessions? (in terms of meeting their needs and in terms of acceptability)	In-depth interviews with health workers. Focus group discussions with health workers. Field notes.
	How effective was the package of interventions in improving health workers’: a. competencies in responding to the SRH needs of adolescent clients, b. attitudes towards meeting these needs, c. motivation to apply their competencies and attitudes to the best of their ability, and d. performance (i.e. clinical and interpersonal practices)?	In-depth interviews with health workers including hypothetical scenarios and role plays. Mystery client assessments. Exit interviews with adolescents and young women. Health worker surveys.

Across all data collection rounds, data will be collected by local research assistants recruited by the University of Kinshasa and trained by the University of Kinshasa and the WHO. All data collection tools were translated from English to French, pilot tested among health workers and health workers at non-participating facilities and as role play simulations for the adolescent tools among research team members and revised in April/May 2021. In-depth interviews and focus group discussions with health worker and health managers will be conducted in French. In the case of mystery clients and adolescent exit interviews, discussions will be conducted in whichever local language the adolescent prefers. Systematic debriefs will take place among the research team after each data collection to ensure richness of the data is not lost. The details of each type of process and outcome data collected are provided below.

### Process evaluation

The objective of the process evaluation is to develop an understanding of the feasibility and acceptability of the package of interventions. The qualitative process evaluation consists of a mix of methods to allow for triangulation, including in-depth interviews, focus group discussions, as well as field notes, monitoring and collaborative learning reports, and costing sheets. The process evaluation consists of an analysis of factors related to intervention package including its delivery (feasibility and cost) and the perceptions of those targeted (adoption and acceptability). This is essential to understand contextual factors affecting the effectiveness of the intervention package and lessons learned in the implementation process in a ‘real world’ setting.

### In-depth interviews and focus group discussion with health workers and health managers

The sampling strategy across all rounds of data collection will be purposive and stratified by health zone to ensure that rural and urban facilities are adequately covered, with the final number of in-depth interviews and focus group discussions determined by data saturation.

In-depth interviews and focus group discussions across all rounds of data collection will consist of a convenience sample of health workers and managers from participating health facilities who have received training and participated in collaborative learning sessions to gain insight into their perspectives and experiences. Specifically, at least two in-depth interviews will be conducted (one with the manager and one health worker at half of participating facilities) for a total of approximately 30 in each round of data collection. Two focus group discussions will be held with health workers in both Kinshasa and Kasai Oriental for a total of four in each round of data collection, to gain insight into attitudes, beliefs, group norms, and dynamics, as well as perceptions of change as a result of the packages of interventions.

The main themes explored in the discussions include: the extent to which the intervention package met their needs, their perceptions, and experiences with the collaborative learning approach, their ability to implement in practice what they learned, and barriers and facilitators that affect their ability to translate their knowledge, skills, and attitudes into practice.

### Field notes

The research team is responsible for taking detailed field notes using a template developed for the project’s data collection manual to ensure standardization and consistency after all data collection activities to document other relevant initiatives in the participating health facilities and communities. This will ensure that a broader understanding of the context can be taken into account during the data analysis process and will assist in understanding attribution of any potential changes in key outcomes to the project.

### Monitoring of implementation

The implementation of the package of interventions will be monitored using a simple reporting form designed to collect information on what was done as well as reflections on the sessions, what went well, and what could be improved in future sessions. Collaborative learning sessions and other monitoring reports will be reviewed during each of the planned rounds of data collection through 2022 to provide information that can be triangulated on evolution of changes, if any.

### Cost analysis

An activity-based costing (ABC) approach will be used to determine how much it costs to deliver the package of interventions, simple cost analysis of the package of interventions will be undertaken to assess feasibility of scale-up, and to inform budgeting for future efforts. This involves a review of documentation from implementing partners RENADEF and Cordaid of the costs of each activity to contribute to an understanding of the cost of each component of the packages of interventions. Initial startup costs for the collaborative learning component of the project have been detailed elsewhere [23].

#### Outcome evaluation

The objective of the outcome evaluation is to assess the effectiveness of the package of interventions in improving health workers’ knowledge, skills, attitudes, and motivation. A quasi-experimental design using survey data collected at four time points, each six months apart will be conducted. Additionally, mystery client assessments and adolescent client exit interviews which will also be conducted at six- month intervals to understand adolescent experiences and perceptions of services received from health workers trained as part of the project.

### Health worker survey

The full details of the validated measures and scales used in the survey will be provided in subsequent publications. The survey was initially piloted in 2021 on a sample of 20 health workers at health facilities not participating in the intervention and revised accordingly. Across each round of data collection, approximately 60 health workers who have received training as part of the project will complete the survey. The survey will be administered to the same sample of participants four more times, at 6-month intervals. Attrition may reduce the numbers of respondents in the follow-up surveys due to staff turnover and other factors; however, efforts will be made to ensure that as many health workers as possible who received training and ongoing support complete the follow-up surveys.

### Mystery client assessments

Mystery client assessments will be conducted in each round of data collection to provide an in-depth understanding of adolescent client experiences of receiving services from health workers supported as part of the intervention. The perspectives of adolescent mystery clients are essential to developing a comprehensive, authentic understanding of how young clients experience receiving health services. This assessment will allow for triangulation with adolescent client exit interviews to assess the perceptions and experiences of adolescents, and the impact of the package of interventions on improving health workers’ performance. The use of mystery clients has been shown to be reliable, valid, feasible, and acceptable for assessing the quality of health services [24].

Ten females between the ages of 18–25 (5 in Kinshasa and 5 in Kasai Oriental) will be recruited by senior members of the research team from a youth organization, the Congolese Youth Association Network (RACOJ). Selection criteria for these positions will emphasize interpersonal skills and professionalism, literacy, field note taking as assessed in a practice session, and non-residence in the vicinity of a participating health facility. Potential mystery clients will be screened to identify those who have previously experienced GBV-related trauma. If they reported they have experienced GBV, they will be referred for counselling and excluded from the selection process to ensure psychosocial harm does not occur as a result of participation in the study. To the extent that it is possible, the same mystery clients will be used in each round of data collection, but they will visit different health facilities in each round.

Mystery clients will receive a 3-day training provided by the University of Kinshasa to conduct the mystery client assessments. The training will be based on approaches that are standardized for training mystery clients, and approaches which were identified as best practices in a related systematic review [24]. Training modules will include sessions on ethics and confidentiality, what to expect in terms of standard of care, how to engage in role play (including rehearsal of possible scenarios), how to ask for information and advice concerning SRH issues, how to conduct observations and memory techniques, and how to complete the checklists and reporting requirements. A refresher training will be provided to mystery clients before each round of data collection.

After training, mystery clients will conduct random visits on different days of the week and different times of day to facilities to enact different scenarios, such as requesting information on STIs including HIV, condoms, or contraceptive methods, or requesting information on how to support a friend who has experienced sexual harassment. They will be given a pseudonym and fictional birth date, and they will carry a letter of participation in case they face any questions about their role in the study. They will be instructed to specify that they are only seeking information, and not to allow any invasive procedures such as pelvic exams or blood tests. A trained staff member from the University of Kinshasa will be available on site when mystery client visits are conducted in case any assistance is needed. The mystery clients will be required to take detailed field notes concerning their visit and participate in a debrief with the local project manager. The assessment and debrief will focus on whether mystery clients are satisfied with the services they received and perceive that they have been treated with respect and empathy and in a non-judgmental manner. Mystery clients will be reimbursed for travel costs, as well as a stipend and per diem for their time for participating in the training and subsequent visits to the health facilities.

### Adolescent client exit interviews

The purpose of the adolescent client exit interviews is to generate complementary data to the mystery client assessments, which will provide an in-depth understanding of adolescent client experiences of receiving services from health workers supported as part of the project.

Only adolescents over the age of 18 years are eligible to participate in exit interviews and in all instances are required to sign an informed consent form. Across each round of data collection, we aim to conduct approximately 10 exit interviews (5 in Kinshasa, 5 in Kasai Oriental) with adolescent clients recruited immediately after they have received information and services at participating health facilities by trained members of the research team.

Key themes explored in the exit interviews with adolescent clients include domains for assessing adolescent-responsive health services in line with the WHO global standards for quality health care services for adolescents, such as equity and non-discrimination [25].

### Triangulation

This mixed-methods study systematically employs quantitative and qualitative methods at all phases of the project and across both the process and the outcome evaluation components. The different types of data to be collected have been selected to carefully align with the objectives of the implementation research, to ensure complementarity, reduce reporting bias, and enhance triangulation and validity of the data. Using different methods will also allow for mitigating the weaknesses associated with relying on one type of evidence. This is particularly important given that satisfaction measures, for instance, may be unreliable as adolescent clients may not be aware of their rights, what constitutes quality care, and in general are subject to social desirability bias. Additionally, a more comprehensive and complete understanding of the packages of interventions will be obtained by ensuring adequate coverage of the range of perspectives about the intervention among key stakeholders.

### Planned data analyses

Qualitative data from in-depth interviews, focus group discussions, mystery client assessments, and exit interviews across all rounds of data collection will be coded thematically based both on *a priori* and emerging themes by two members of the research team to ensure quality control and consistency in coding in Atlas.ti. Relevant constructs from the cluster of theories that underpin the design and evaluation of the project will also be used when analyzing in-depth interviews and focus group discussions with health workers and health managers to shed light on the process of change in the provision of services to adolescents as a result of the intervention. Among health workers and health managers, themes to be explored include: knowledge, skills, and attitudes related to the provision of SRH services to adolescents; facilitators, and barriers to providing quality care to adolescents including the provision of care to those reporting having experienced violence; factors influencing health worker motivation, and performance; and perceptions of relevance and adequacy of the support provided as part of the Project. From the adolescent client perspective (both mystery clients and exit interviews), the analysis will focus on whether they perceive that they were treated with respect, sensitivity, dignity, and empathy. Qualitative data will be transcribed in the language in which the discussion was conducted and translated into French if necessary. The accuracy of the translations will be verified independently through a process of back-translation.

Survey data will be exported from the ODK tablets to Excel and after verification exported to Stata 14 analysis software. Statistical analyses will consist of descriptive statistics including frequency tables, as well as bivariate analyses to classify frequency distributions of attitudes and practices according to knowledge and skills levels. Logistic regression and time series analyses may be used to examine associations and look at changes over time.

### Stakeholder engagement and dissemination

During the evaluation phase relevant stakeholders at the local and national level such as the Ministry of Health through PNSA, PNLS and health zone management teams, and local civil society bodies representing adolescents including RACOJ, RENADEF, and CORDAID will be involved in a Technical Advisory Committee (TAC) to ensure widespread support and understanding of the project activities and main findings.

The findings will be disseminated in national and international fora and at relevant policy-level events to ensure that the results promote policy dialogue and improve the health sector response to the provision of services and care to adolescents.

### Ethical considerations

The study adheres to the ethical and safety guidelines laid out in the *Standards and operational guidance for ethics review of health-related research with human participants* [26] and the *WHO guidance on ethical considerations in planning and reviewing research studies on sexual and reproductive health in adolescents* [27]. The protocol has been approved by the WHO’s Ethical Review Committee and the University of Kinshasa’s Ethics Review Board.

All participants including adolescents (all of legal age of majority i.e. over 18 years) participating in mystery client assessments, adolescent exit interviews, as well as health managers and health workers participating in in-depth interviews, focus group discussions, and completing the survey, are required to provide written consent. Mystery client assessments, adolescent exit interviews, in-depth interviews and focus group discussions will be conducted in a private setting to be set up at the health facilities.

Participants will be informed about the confidentiality procedures as part of the consent process. To ensure confidentiality, a code is assigned to each participant instead of individuals’ names on data collection tools, in transcripts and reports. No information that can be used to identify individuals will be transcribed or reported (for example indirect identifiers such as names of other persons mentioned, place names etc.). Any information, including potentially sensitive information about participants will be kept confidential and data analyses will be done on de-individualized samples. An electronic master list of names and codes will be managed by the PIs of the project. Hard copies of consent forms will be kept in a secure, locked location at Kinshasa School of Public Health. All transcripts, translations, field notes, and databases will be locked either physically or electronically, with only members of the research team able to access data. All confidential materials will be accessible only to senior members of the research team. Audio-recordings will be deleted once transcripts and reports summarizing the findings have been finalized. In-depth training has been given to data collectors on issues related to confidentiality and providing referrals in case of distress and refresher training will be provided prior to each round of data collection.

## Discussion

The forthcoming findings of this implementation research will shed light on the perspectives and experiences of health workers and health managers who have participated in the study, and adolescents who have received services from health workers trained through the project. We anticipate the findings will also contribute to the growing body of evidence of what works in terms of improving health workers’ knowledge, skills, and attitudes in providing sexual and reproductive health services to adolescents. In particular, the study will generate findings on the feasibility, acceptability and effectiveness of the collaborative learning model as an approach to train health workers (which could be relevant for other target groups such as schoolteachers) to jointly identify, define and solve issues related more broadly to adolescent health issues ranging from non-communicable diseases (NCDs), to communicable diseases and a host of other issues such as early/unwanted pregnancy, violence and mental health.

This implementation research will contribute new knowledge related to several of the Sustainable Development Goals (SDGs) and their achievement, for instance the crucial role of the health workforce in delivering on SDG goal 3 (health and well-being) and SDG goal 5 (gender equality) in particular. Competent and motivated health workers deployed in the right places in adequate numbers, receiving full support from health authorities and partners are necessary for a strong primary health care system and for making progress towards universal health coverage, and specifically for promoting SRH and preventing early pregnancies, and sexually transmitted infections including HIV infection, and detecting and responding to challenges when they occur [28]. This forthcoming findings from this study may also contribute to progress related to SDG goals 3 and 5 by generating evidence of what works and in which settings in terms of improving and maintaining improvements in health worker knowledge, skills, and positive attitudes towards the provision of sexual and reproductive health care and services to adolescents. Developing and rigorously evaluating a package of interventions to train and support health workers and create an enabling environment for them to apply their competencies and attitudes and evaluating the effectiveness of these interventions on key outcomes, is crucial in efforts to improve health and well-being [29].

Additionally, the study findings may also contribute new knowledge which addresses the issue of SRH of adolescents, a cross-cutting theme in the SDGs, through targets on ensuring universal access to services, reducing the number of early pregnancies, new HIV infections, eliminating violence against women and girls, and reducing maternal mortality. The inclusion of mystery client assessments and exit interviews with adolescent clients will ensure their perspectives and experiences of receiving health care and services from trained providers are taken into consideration in the planning of future interventions and programmes.

Finally, this study, which aims to systematically apply a cluster of interrelated theories from social and behavioral science, may contribute to advancing understandings of how new practices and norms become adopted in health facilities. This is particularly relevant given the study’s focus on introducing the collaborative learning approach. The application of a theoretical lens may also assist in identifying barriers to uptake and provide important insights for potential future adaptation and scale-up of the package of interventions to other settings.

We do however acknowledge that there are limitations to our study. Firstly, this implementation research is nested within an ongoing initiative which did not have an evaluation plan built into the project at the outset. This also presents a unique learning opportunity however, since it reflects the reality of many similar projects, and we will be able to demonstrate that it is possible to establish a robust process and outcome evaluation in the midst of implementation. Secondly, the study is only conducted in two provinces in the DRC which may limit the generalizability of findings to other contexts and areas in the country. On the other hand, there is one urban area and one rural area represented. Thirdly, the sample size of health workers completing the survey is relatively small thereby limiting our statistical power. However, we have enrolled every health worker who is a part of the initiative which will provide a comprehensive overview of participant perspectives in the project. An additional limitation related to the study design is that the implementation research lacks a comparison group which hinders our ability to demonstrate a causal relationship between the intervention and any outcomes. To address this shortcoming, we may consider inclusion of a comparison group in subsequent rounds of data collection. Finally, as with any quality improvement initiative, effects and sustainable change tend to be slow to emerge and may be affected by health worker or health manager turnover, competing priorities in a complex health system, and other contextual factors. Nevertheless, our mixed-methods time series design will allow us to triangulate data collected using different methods to reduce bias, as well as rigorously monitor change and document information on any other initiatives aside from the intervention that may influence reported outcomes and provide important information for future studies and efforts to scale-up.

## Conclusions

Interventions which aim to move beyond one-off trainings and didactic approaches and towards comprehensive and collaborative learning approaches to improve health workers’ knowledge, skills, and attitudes in providing sexual and reproductive health services to adolescents, alongside creating an enabling work environment are urgently needed. We anticipate that the findings from this mixed methods time-series implementation research will be useful in subsequent adaptations and/or scale-up of the package of interventions to improve health worker motivation and performance in the Democratic Republic of the Congo and beyond. More broadly, the findings from the study will also contribute to evidence base of how to apply theoretical approaches within the field of implementation research.

## Paper context

There is limited knowledge of how to deliver effective interventions at scale in resource constrained settings. This study describes the development, implementation, and evaluation plan to assess the feasibility, acceptability, and effectiveness of a package of interventions aimed at improving health worker motivation and performance in the Democratic Republic of the Congo. The findings will contribute to the evidence base of how interventions can move beyond piecemeal efforts and towards comprehensive approaches.
